# Modeling and simulation of biological systems using SPICE language

**DOI:** 10.1371/journal.pone.0182385

**Published:** 2017-08-07

**Authors:** Morgan Madec, Christophe Lallement, Jacques Haiech

**Affiliations:** Laboratory of Engineering Sciences, Computer Sciences and Imaging (ICube, University of Strasbourg / National Center for Scientific Research), Solid and Systems Electronic Department, Strasbourg, France; Lanzhou University of Technology, CHINA

## Abstract

The article deals with BB-SPICE (SPICE for Biochemical and Biological Systems), an extension of the famous Simulation Program with Integrated Circuit Emphasis (SPICE). BB-SPICE environment is composed of three modules: a new textual and compact description formalism for biological systems, a converter that handles this description and generates the SPICE netlist of the equivalent electronic circuit and NGSPICE which is an open-source SPICE simulator. In addition, the environment provides back and forth interfaces with SBML (System Biology Markup Language), a very common description language used in systems biology. BB-SPICE has been developed in order to bridge the gap between the simulation of biological systems on the one hand and electronics circuits on the other hand. Thus, it is suitable for applications at the interface between both domains, such as development of design tools for synthetic biology and for the virtual prototyping of biosensors and lab-on-chip. Simulation results obtained with BB-SPICE and COPASI (an open-source software used for the simulation of biochemical systems) have been compared on a benchmark of models commonly used in systems biology. Results are in accordance from a quantitative viewpoint but BB-SPICE outclasses COPASI by 1 to 3 orders of magnitude regarding the computation time. Moreover, as our software is based on NGSPICE, it could take profit of incoming updates such as the GPU implementation, of the coupling with powerful analysis and verification tools or of the integration in design automation tools (synthetic biology).

## Introduction

SPICE (Simulation Program with Integrated Circuit Emphasis) is a general-purpose analog electronic circuit simulator developed at the Electronics Research Laboratory of the University of California, Berkeley by Laurence Nagel in 1970 [1]. As its name suggests, SPICE is dedicated to the design of large scale integrated circuit (IC) and printed circuit boards, to the checking of circuits integrity and to the prediction of circuits behavior. In the past 40 years, SPICE “*evolved to become the worldwide standard integrated circuit simulator*”, as stated in an IEEE Milestone given to this software in 2011. The first commercial SPICE simulator was released in the 80s. Nowadays, the most common ones are PSPICE owned by CADENCE Design Systems and HSPICE owned by Synopsis. Alternatively, there is also a plenty of open source SPICE distributions that have mostly been developed by and for academic research: XSPICE [2], NGSPICE [3], LTSpice, TinySPICE [4], etc

The program takes as an input a textual netlist describing the assembly of elementary electronic devices (resistors, capacitors, voltage and current sources, transistors, etc) connected together on electrical nodes. It translates this list into a set of ordinary differential equations (ODE) with respect to the electrical Kirchhoff laws. The netlist is very simple. This is probably one of the main asset that contributed to the democratization of this language in the industry of microelectronics. Basically, each line of the netlist corresponds to the instantiation of one device. For each, a label which first letter determines the type of device, a list of the nodes to which the device is connected and a list of the parameters of the model are provided [5,6]. For instance, the line R1 A B 100 means that a 100-ohm resistor between nodes A and B. Thus, a SPICE netlist is human-readable, can be easily interpreted by a software and can be easily generated from an electronic schematic or from any other software. Present versions of SPICE involve many simulation and analysis capabilities including steady state (operating point) analysis, transient analysis, linear small-signal frequency domain analysis, parametric sweep, noise analysis, transfer function analysis, pole-zero analysis, stability calculation, etc. SPICE also allows parameterization of design variables to accelerate design automation of integrated circuits and their optimization, as shown [7].

Although SPICE was originally a software dedicated to the design of electronic circuits, it has already been extended to other fields of physics, such as thermal and electro-thermal simulations [8,9], optics and optoelectronic devices [10,11], mechanical systems [12] and microfluidics [13]. It has also been used to simulate the dynamics of biological systems whose description is based on differential equations [14–16]. We propose, in this paper, a way to formalize this approach.

In systems biology, description and simulation software tools appeared much more recently. The first and most successful effort in this domain is probably the System Biology Markup Language (SBML) [17]. SBML is dedicated to the description of a biochemical system (such as a gene regulatory network, a metabolic pathway, etc). A SBML description is an XML file which contains among other a list of parameters, a list of involved chemical species and a list of reactions with, for each, a rate equation. Most of the time, the XML file is generated through a graphical user interface such as SBMLEditor or Virtual Cell [18] or picked up from databases such as BioModels [19]. Nevertheless, due to its relative complexity, SBML models are not easy to read and edit by a user or a third-party software. This is not very convenient for applications where the model is extensively manipulated. SBML description can be handled by dedicated tools to simulate the behavior of the system. COPASI (Complex PAthway Simulator), developed in 2006 by Hoops et al, is becoming the reference in this domain [20].

Recently, we demonstrated an analogy that can be drawn between the behavior of biochemical systems and electronic circuits [21]. This analogy is based on the notion of ‘biological transistor’. Indeed, an electronic transistor can be seen as a current source (*i*.*e*. source of electrons) controlled by a voltage. These devices are modeled by a variable conductance, or a voltage-controlled current sources (VCCS). Similarly, a chemical reaction can be seen as a source of molecule (positive or negative) controlled by the concentration of other molecules. Consequently, the analogy between concentration and voltage on the one hand, and molecule flow and electrical current on the other hand makes possible the simulation of biological with SPICE. More details about this analogy are given in the Method section. This approach is mainly motivated by two outcomes: the development of design tools for synthetic biology and the virtual prototyping of biosensors and lab-on-chip.

First, synthetic biology is a new domain of the biotechnologies which aims at reinvesting system’s engineering methods and biological knowledge in order to design new biological function by assembly of standardized parts [22]. *In silico* design tends to play a major role in synthetic biology and since about 10 years, many computer-aided design tools have been developed for such purpose. Most of the time, they have been developed from scratch [23–27]. An alternative approach, which have been recently demonstrated, is to use and readapt existing tools from the domain of microelectronics (Electronic Design Automation or EDA) to synthetic biology [21,28]. As simulation is at the heart of EDA processesa prerequisite for the adaptation of EDA tools to biology is the possibility to simulate biological mechanisms with SPICE.

Second, there are more and more applications with a direct coupling between a biological or biochemical systems and an electronic devices: bio-sensor, lab-on-chip, live cell-based sensor, DNA chips, etc [29]. Design automation of such systems requires a unique description language and simulation environment that encompasses all the disciplines involved in such system (electronics, fluidics, mechanics, biology …). It has already been shown that SPICE could be extended to the majority of physical disciplines [8–16]. An extension of SPICE to biology is the next logical step to go toward this unique environment.

The idea behind BB-SPICE (SPICE for Biology and Biochemistry) is to offer a way to describe biological functions in a SPICE-like netlist and simulate it with a SPICE distribution. The other requirements for the targeted tool are: i) to be open-source, ii) to be usable by biologists without any knowledge on electronics, on the SPICE language or on the associated simulator and iii) to keep upstream and downstream compatibility with SBML (*i*.*e*. a SBML model must be able serve as simulation input file in replacement of the SPICE-like netlist and SBML model must be generated from a BB-SPICE netlist. This last specification is required in order to keep benefits of research efforts in SBML and associated tools and databases. For the present work, the compatibility with SBML tools is also mandatory in order to compare both approaches in terms of computation time and simulation accuracy.

## Methods

**Fig 1:**
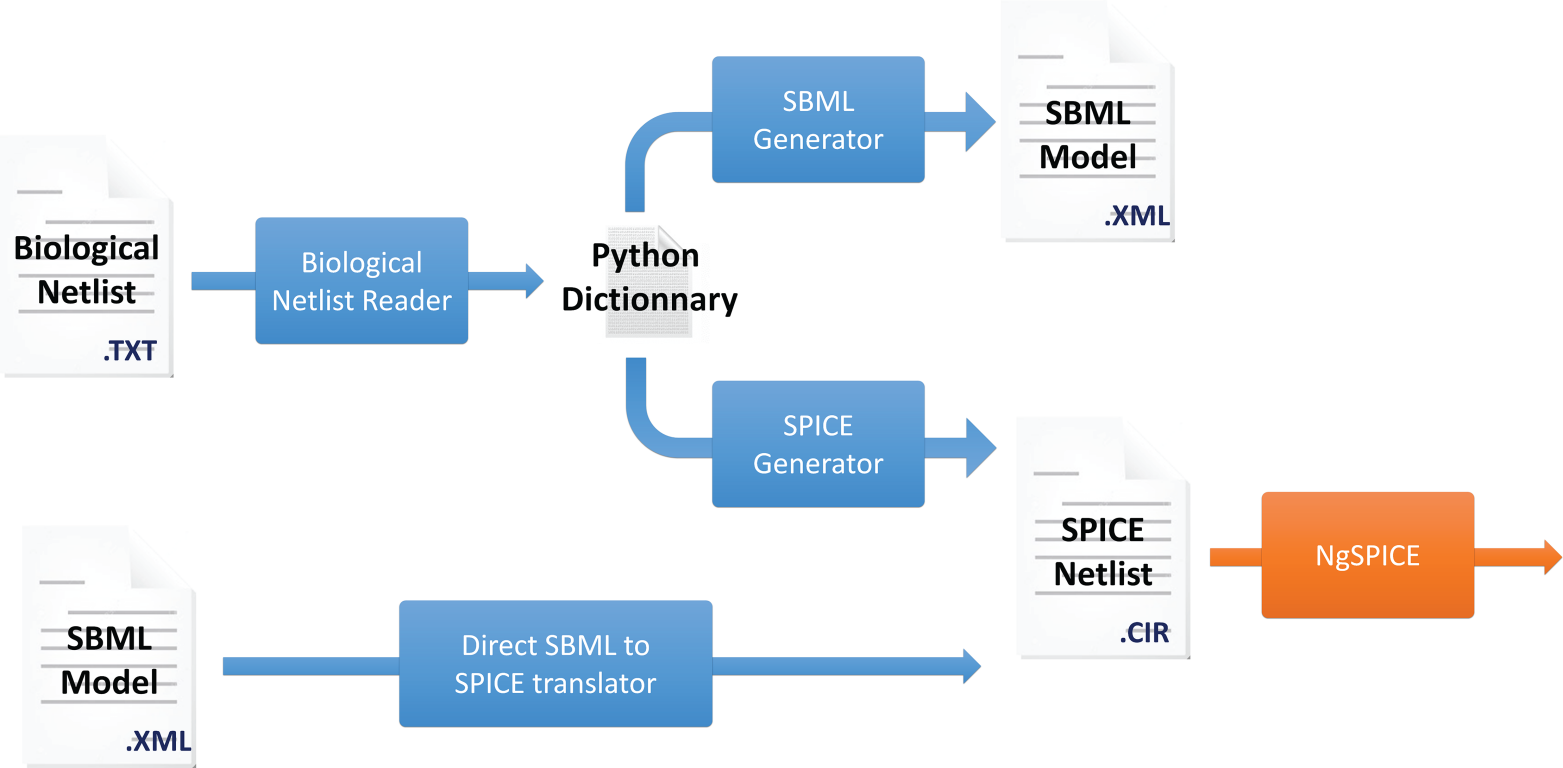
The BB-Spice environment. The software environment is composed of 5 tools: a BB-SPICE netlist parser, a SPICE netlist generator, a SBML-to-SPICE translator, a SBML model generator and a SPICE simulator, *i*.*e*. NGSPICE.

BB-SPICE is composed of five modules, as depicted in Fig 1.

- A biological netlist parser which takes as input a text file with the description of the biological system in a formalism described hereafter. The parser generates a Python dictionary which contains the list of species, the list of reactions, the reaction parameters, etc.
- A SBML file reader which also generates a Python dictionary for a given biological system.
- A SBML file generator, which takes as input the Python dictionary described hereabove and generates an SBML description.
- A SPICE file generator, which takes as input the Python dictionary described hereabove and generates a SPICE netlist.
- A open-source SPICE circuit simulator, NGSPICE in this particular case [3].

More details on the building blocks are given in the Translators subsection.

### Background: Equivalence between biological systems and electronic circuits

An analogy can be drawn between a biological system and an equivalent electronic circuit [21]. Basically, this analogy consists in considering molecules as electrons. Thus, a (positive or negative) source of molecules is equivalent to a source of electrons (current source) in electronics. Moreover, the local concentration of a molecule in a point can be seen as an accumulation of molecules at this point, just as electrons in a capacitor. This accumulation of molecules can be modeled by a capacitor which value depends of the volume in which molecules are accumulated. From a kinetic viewpoint, any chemical reaction can be considered as a positive or a negative sources of molecules which value (production/consumption rate) depends on the concentration of other involved or third-party molecules. According to our analogy, any chemical reaction can be modeled as a set of current sources controlled by other voltages. This behavior reminds the behavior of electronic transistor. Thus, the symbol of a BJT transistor is used to depict these sources of molecules in the electronic equivalent representation of a biochemical system. In practice, these “biological transistors” are modeled by variable conductances or VCCS which value corresponds to the rate equation of the reaction. The main difference between electronics and biological circuits is that the information carrier is unique in electronic and multiple in biology (each species may carry information). As a consequence, the ports of the models of biological device have to be tagged by the species name. Putting together all biological equivalent devices leads to an electrical network composed of several VCCS, possibly resistors which model the natural degradation of the molecule and capacitors which model the accumulation of molecules at a given point of the space.

**Fig 2:**
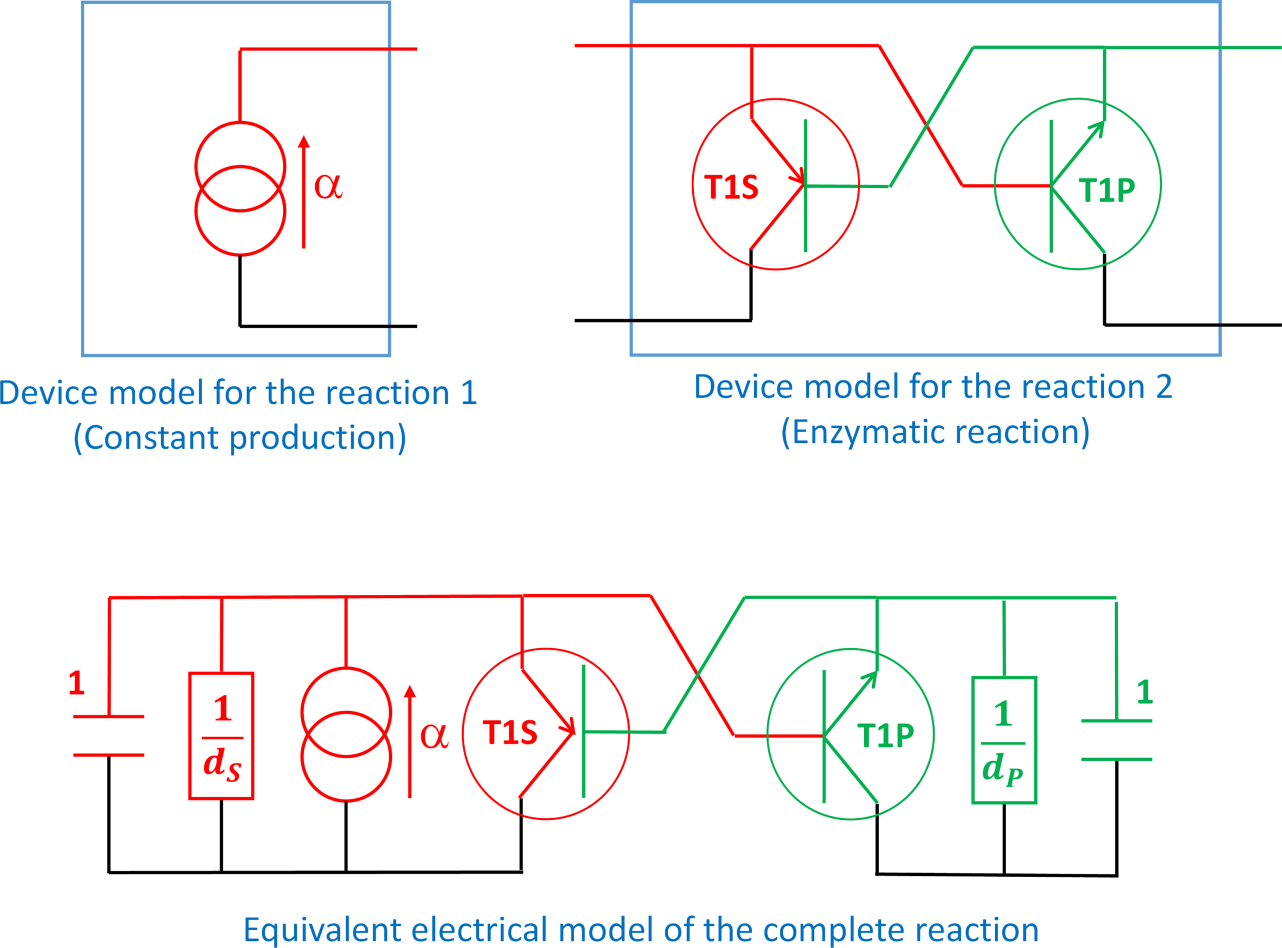
Electrical equivalent circuit for the self-inhibited enzymatic reaction. The sub-circuit (A) corresponds to the model of the constant production of the substrate. The sub-circuit (B) is the model of the enzymatic reaction. It is composed of a biological transistor modeling the synthesis of the product and which depends on the concentration of substrate and product according to Eq (3) and another biological transistor modeling the consumption of the substrate which also depends on the concentration of the substrate and the product. (C) corresponds to the complete electrical equivalent circuit obtained by putting together sub-circuits (A) and (B) and adding degradation resistors and capacitors.

The principle of the equivalence is illustrated hereafter on two examples. The first one is a self-regulated enzymatic reaction. Consider two molecules S and P and two chemical reactions: i) the constant production of S at a given rate *α* and ii) an enzymatic reaction inhibited by P and modeled by a modified Michaelis-Menten’s law [30] including a Hill-like inhibition term depending of P [31]. The ordinary differential equations (ODE) that govern this system are the following:

$$\frac{d[S]}{dt}=\alpha -\frac{V_{max}∙[S]}{K_{m}+[S]}∙\frac{{K_{P}}^{n_{B}}}{{K_{P}}^{n_{B}}+{\left[P\right]}^{n_{B}}}-d_{S}∙[S]$$ (1)

$$\frac{d[P]}{dt}=\frac{V_{max}∙[S]}{K_{m}+[S]}∙\frac{{K_{P}}^{n_{B}}}{{K_{P}}^{n_{B}}+{\left[P\right]}^{n_{B}}}-d_{P}∙[P]$$ (2)

with *V*_*max*_ the maximal rate of the enzymatic reaction, *K*_*m*_ the Michaelis constant, *K*_*P*_ and *n*_*P*_ the Hill constant and the Hill number for the inhibition term and *d*_*S*_ and *d*_*P*_ the natural degradation rate for S and P. The electronic equivalent circuit for this system is given in Fig 2. On the one hand, the constant production of S is modeled by a constant current source. On the other hand, the enzymatic reaction is modeled by two biological transistors T1S and T1P which conductance is:

$$G_{T1P}={-G}_{T1S}=\frac{V_{max}∙[S]}{K_{m}+[S]}∙\frac{{K_{P}}^{n_{B}}}{{K_{P}}^{n_{B}}+{\left[P\right]}^{n_{B}}}$$ (3)

Putting together both devices and adding degradation resistors and capacitors leads to the electrical circuit given in Fig 2. Application of Kirchhoff laws to this circuit (with *V*_*x*_ ≡ [*x*]) leads to the same ODE set as (1) and (2).

**Fig 3:**
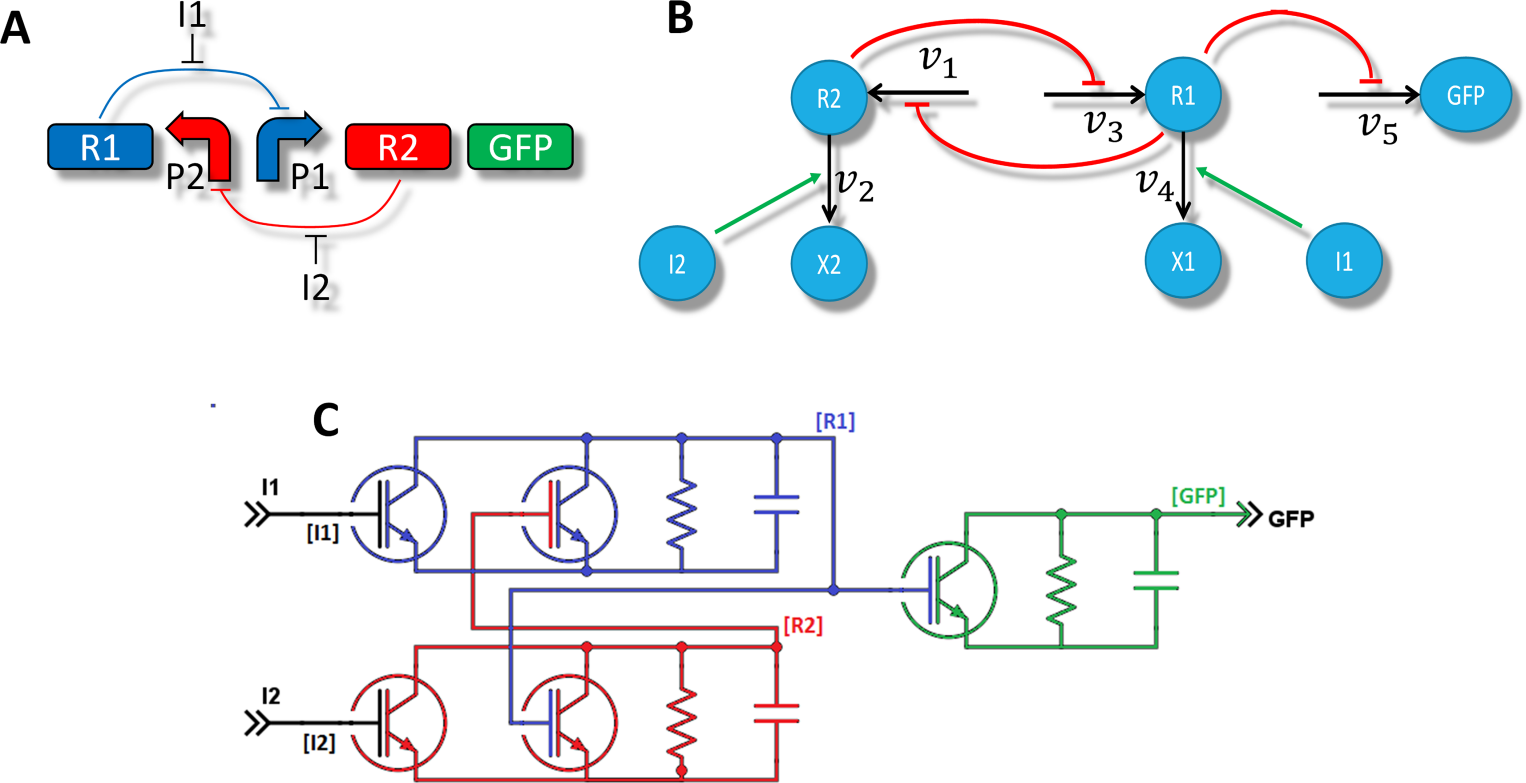
Electrical equivalent circuit for the toggle switch. (A) is the biological representation of the system as regulated genes. (B) is the interaction network representation of the toggle switch and (C) is the electronic equivalent circuit. Nodes X1 and X2 as well as associated devices are not represented in the schematic for simplicity sake.

The second example is a genetic toggle switch [32] described by Fig 3. This system is composed of 5 reactions and 7 species. Reaction 1 is the production of molecule R2 which can be inhibited by another molecule R1. Reaction 2 is the production of molecule R1 which, in turn, can be inhibited by the molecule R2. Reaction 3 is an enzymatic reaction that transforms R2 into X2. It is catalyzed by an enzyme I2. By the same way, Reaction 4 is also an enzymatic reaction catalyzed by I1 that transforms R1 into X1. Finally, Reaction 5 is the production of a green fluorescent protein (GFP) which is inhibited by R1. The network of interaction associated with this system is given in Fig 3B. The toggle switch has two stables steady states: the state “1” in which R2 and GFP are produced and the state “0” in which R1 is produced. Switching from one state to another is possible by injection of I1 or I2. For instance, if the system is in state “1” and I1 is injected, R1 is consumed by the enzymatic reaction 4. Thus, the production of R2 is no longer inhibited and the system reach the state “0”. The electrical equivalent circuit of this system is composed of 5 nodes and 5 biological transistors (Fig 3C). The concentrations of X1 and X2 have no influence on the behavior of the system. Thus, for simplicity sake, the nodes X1 and X2, as well as the devices associated with these nodes, are not represented. The model behind the transistors T1, T2 and T5 corresponds to the constant production with a Hill-like inhibition term [31] whereas the model behind T3 and T4 corresponds to a standard Michaelis-Menten equation [30].

### ngSPICE

NGSPICE is an open-source circuit simulator based on SPICE 3f5. It includes software tools for the parsing of netlists and for the computation of steady state, transient and frequency analysis. Moreover, NGSPICE integrates a simple but efficient scripting language that can be used to program complex simulation processes for a given system (*e*.*g*. computation of the steady states for different sets of parameters, computation of transient simulation with different inputs, statistical analysis, etc).

### BB-SPICE netlist format

A BB-SPICE file looks like a SPICE netlist but contains the same information as in a SBML model. It is composed of 4 sections: parameters definition, species definition, reactions definition and simulation directives. Inside each section, the description of the species, the parameters and the reactions are made according to a SPICE-like format, *i*.*e*. a raw text file with one line per instance and argument separated by blank characters. For parameters, user has to define a name, a value and an optional comment. For species, user has to define a name followed by up to two arguments which correspond respectively to the initial concentration (default 0) and the natural degradation rate (default 0). Finally, for each reaction, as in NGSPICE, user gives a reaction name followed by 1 to 4 arguments that may have different significations depending on the reaction type. The first letter of the reaction name indicates its type. The last argument is always the set of parameters to use to parametrize the generic rate equation associated with the reaction type. The list of the reaction types that are implemented in present version of BB-SPICE is described in Table 1. Whatever the argument, when multiple values are required (*e*.*g*. enzymatic reaction requires two parameters, one for *V*_*max*_ and one for *K*_*m*_), they should be given as a list separated by comma and between braces. Conversely, if no value is required for a given argument (*e*.*g*. a transcription which is not regulated), the argument should be 0. The fourth part of the BB-SPICE netlist concerns the simulation directives. It corresponds to pre-defined stimuli (*e*.*g*. fixed concentration for a given species, constant, pulsed or periodic molecule sources, etc), the analysis to perform (transient, steady-state, frequency analysis, etc) and the results to plot. These directives are translated into devices and/or NGSPICE control instructions. They are summarized in Table 2.

**Table 1:** Types of reactions implemented in the current version of BB-SPICE.

First letter	Reaction type	Argument #1	Argument #2	Argument #3	Parameters to provide^a^
B	Binding reaction	Reactants	Products		*k*_*on*_ and *k*_*off*_
C	Constant production	Products			*β*
D	Simple decay	Degraded Species			*d*
E	Enzymatic reaction	Substrates	Products	Enzymes	*v*_*max*_ and *K*_*m*_
F	Passive diffusion	In Species	Out Species		*D*
H	Hill-like controlled reaction	Reactants	Products		*v*_*max*_, *K* and *n*
L	mRNA translation	Proteins	mRNAs		*k*_*TL*_
P	Pump	In Species	Out Species	Modifiers	*v*_*max*_ and a couple of Hill’s (*K*_*j*_ and *n*_*j*_) per regulating protein
T	DNA Transcription	mRNAs	Activators	Repressors	*k*_*TR*_, *KA*_*j*_ and *nA*_*j*_ for activators, *KR*_*j*_ and *nR*_*j*_ for repressors
X	Custom reaction	Reactants	Products	Modifiers	The reaction rate between simple quotes

List of the 9 regular reaction that are supported by the current version of BB-SPICE. For each of them, 1 to 4 argument have to be given, depending on the reaction type. They are described in the third, fourth and fifth column of the table. The last argument to provide is the list of parameters which also depends on reaction type. These parameters are described in the last column of the table. Finally, the last line is the custom-rate reaction for which, the reactant, the products, the modifier and the reaction rate have to be provided.

^a^Parameters given in the last column correspond to parameters described in Eqs (4) to (10).

**Table 2:** List of the simulation directives implemented in the current version of BB-SPICE.

Directive	Type	Interpretation
FIX	Stimulus	Fixes the concentration of a species to a given value
PULSE	Stimulus	Defines pulsed source of molecules for a given species. Parameters: delay, pulse length, pulse height, frequency)
WAVEFORM	Stimulus	Generates a source of molecules composed of piecewise constant values for the given species
TRANSFUNC	Stimulus Analysis	Computes the steady state of the system and plots the Bode diagram of the system around this steady state.
TRUTHTABLE	Stimulus and Analysis	Generates automatically a set of periodic on-off sources of molecules for several species in order to cover all the combinations from a Boolean viewpoint.
STEADY	Analysis	Computes the steady state of a system
SWEEP	Analysis	Computes the steady state of the system when one input vary in a given range
TIMECOURSE	Analysis	Computes the transient evolution of a system, starting from the initial condition defined for each species.
PLOT	Control	Plots the concentration of selected species as a function of the time or another concentration (depending on the analysis).
PLOTALL	Control	Plots the concentration of all species involved in the system as a function of the time or another concentration (depending on the analysis).

List of the 10 simulation directives supported by current version of BB-SPICE. They are three kind of directive (second column): stimuli, analysis and control. They are described in more details in the last column.

### Associated models

This section describes the models used for the biological mechanisms described in Table 1. For the binding reaction, a first-order reaction rate is used. Let R_1_, … R_N_ be the reactants and P_1_, … P_M_ be the products. The reaction rate depends on the stoichiometry coefficient for each reactant (*nR*_*k*_) and each product (*nP*_*k*_) as well as two parameters: the forward reaction constant *k*_*on*_ and the reverse reaction constant *k*_*off*_ which is equal to zero if the reaction is irreversible. The rate equation is the following:

$$v_{B}=k_{on}∙\prod_{k=1}^{N}{\left[R_{k}\right]}^{{nR}_{k}}-k_{off}∙\prod_{k=1}^{M}{\left[P_{k}\right]}^{{nP}_{k}}$$ (4)

For enzymatic reactions, a Michaelis-Menten model is used [30]. Let S be the substrate and E the enzyme. The reaction rate is given by:

$$v_{E}=\frac{v_{max}∙[E]∙[S]}{K_{m}+[S]}$$ (5)

where *v*_*max*_ is the maximal reaction rate per enzyme and *K*_*m*_ the Michaelis constant.

The passive diffusion of a given species X between two points A and B of the space is based on a simple linear relationship with the gradient of concentration. It only depends on a diffusion constant *D*. The species at the point A is consumed with a rate *v*_*D*_ whereas the species in B is produced at this rate.

$$v_{D}=D∙({\left[X\right]}_{A}-{\left[X\right]}_{B})$$ (6)

In practice, the two VCCS in A and B can be replaced by a single resistor between A and B and with value of 1/*D*.

As its name suggests, the Hill-like controlled reaction is modeled by a Hill equation [31]. Let R be the reactant and P the product of the reaction. The reaction rate depends on the concentration of reactant as well as three parameters: the maximal reaction rate *v*_*max*_, the Hill’s constant *K* and the Hill’s number *n*.

$$v_{H}=\frac{v_{max}∙{\left[R\right]}^{n}}{K^{n}+{\left[R\right]}^{n}}$$ (7)

mRNA translation rate is modeled by a linear relationship with the mRNA concentration:

$$v_{L}=k_{TL}∙[mRNA]$$ (8)

Where *k*_*TL*_ is the translation rate per mRNA,

A pump transfers a given species X from a point A to a point B. By opposition with the passive diffusion, the transfer (reaction) rate does not depend on the gradient of concentration but is regulated by the concentration of X in A and/or B and/or by third-party molecule. Regulations are modeled by Hill’s terms [33]. Let M_1_, … M_N_ be the regulating molecules The reaction rate is given by:

$$v_{P}=v_{max}∙\prod_{j=1}^{N}\frac{{\left[M_{j}\right]}^{n_{j}}}{K_{j}^{n_{j}}+{\left[M_{j}\right]}^{n_{j}}}$$ (9)

where *v*_*max*_ is the maximal pumping rate, *K*_*j*_ is the Hill’s constant associated with the regulating molecule M_j_ and *n*_*j*_ is the Hill’s number for the same regulating molecule.

DNA transcription is also modeled by a maximal transcription rate (*k*_*TR*_) modulated by a Hill-like regulation term. Let A_1_, … A_N_ be the transcription factors that activate the transcription and R_1_, …, R_M_ the transcription factors that inhibit it. The regulated transcription rate is given by:

$$v_{T}=k_{TR}∙\frac{\sum_{j=1}^{N}{\left(\frac{\left[A_{j}\right]}{{KA}_{j}}\right)}^{{nA}_{j}}}{1+\sum_{j=1}^{N}{\left(\frac{\left[A_{j}\right]}{{KA}_{j}}\right)}^{{nA}_{j}}+\sum_{j=1}^{M}{\left(\frac{\left[R_{j}\right]}{{KR}_{j}}\right)}^{{nR}_{j}}}$$ (10)

where *KA*_*j*_ and *nA*_*j*_ are the Hill’s constant and Hill’s number associated with the activator A_j_ and *KR*_*j*_ and *nR*_*j*_ are the Hill’s constant and Hill’s number associated with the activator R_j._

The models for a constant production and a simple degradation are obvious. Finally, for a user-defined reaction, the equation rate is directly hardcoded as an argument in the netlist.

### Translators

#### BB-SPICE parser

The BB-SPICE netlist is read by a Python script and converted into a set of dictionaries that contain all the relevant information about the system. There is one dictionary for the parameters, one for the species and one for each type of reaction. The simulation directives are also listed.

#### NGSPICE netlist generator

The NGSPICE netlist is generated from the set of dictionaries. First, model parameters are defined as generic parameters (.param) in the NGSPICE netlist. Parameters may depend on other parameters defined previously. Obviously cyclic parameters definition has to be avoided and returns errors during the compilation of the generated NGSPICE netlist.

According to the Figs 2 and 3, the definition of a species in the netlist corresponds to the definition of an electrical node with a capacitor and a resistor in the electronic equivalent circuit. The name of the electrical node will be the name of the species. The natural degradation rate of the species is used to fix the value of the resistor and its initial concentration is used to fix the initial voltage (ic) across the capacitance. Again, these two arguments may depend on user-defined parameters.

Each element of the reaction list is transformed into a resistor (Rx), a current source (Ix) or a custom conductance (Gx) according to rules that depends on the associated reaction. Generally speaking, a reaction with *N* reactants and *M* products is converted into *N*+*M* user-defined non-linear VCCS instantiated between the node corresponding to each species and the ground. The equation of the VCCS is the reaction rate. The directions of the source are opposite for a reactant and for a product. The only exception concerns the diffusion which is modeled by a single resistor, the linear degradation which is modeled by a resistance connected to the ground and the constant production which is modeled by a fixed current source.

Finally, simulation directives are transposed into devices, NGSPICE simulation directives and control statements. For instance, the FIX directive statement is converted into a fixed voltage source (Vx). PULSE and TRUTHTABLE directives statements leads to the implementation of pulsed current sources (IPULSEx), etc. TIMECOURSE and TRUTHTABLE directives are transformed in.TRAN simulation directive, SWEEP in.DC simulation directive and TRANSFUNC in.AC simulation directive. Finally, PLOT and PLOTALL are transformed into the equivalent NGSPICE control statements.

In addition to predefined features described in Table 2, user may define more sophisticated stimulus, instantiate extra devices or perform other analysis (e.g. parametric sweep, noise analysis …). Associated code lines have to be written directly in NGSICE language and preceded by the character $. These lines are echoed in the generated NGSPICE file.

#### SBML importer

The SBML model importer relies on the Python libSBML module [34]. Relevant information picked out of the model are recorded in dictionaries. First, electrical devices are implemented according to several rules. Then, the model is translated into electronic devices (capacitors, VCCS, voltage sources …) according to several rules. Basically, a SBML model is composed of fixed parameters, species and reactions with associated rates. Translation of parameters is straightforward. Then, as for BB-SPICE model, a capacitor is instantiated for each species. Finally, for each reaction and each species involved in the reaction, a positive (resp. negative) VCCS is instantiated. The value of the VCCS corresponds to the formula encoded in MathML inside the SBML model.

Several specific features of SBML language that are supported by the current version of the translator are the following:

- Parameters which scope is limited to a given reaction rate are not implemented but substituted by their actual value directly in reaction rate equations.
- Assignment rules are implemented as voltage controlled voltage sources.
- Rate rules are implemented as voltage controlled current sources.
- Compartments are taken into account: the value of the capacitor associated to each species depends on the size of the compartment.
- User-defined functions are also translated. As NGSPICE does not provide any mechanism for function definition, a dedicated libSMBL method is used in order to substitute each invocation of the function found in formulas by a in-line copy of the function's body.
- Events that affect parameters and depend only on time are also implemented. Affected parameters are transformed in nodes with an associated conditional voltage source.

Conversely, other features such as units, events that depend on species concentration or modify species concentration and algebraic rules are not yet supported.

#### SBML generator

A SBML model can also be generated from the set of dictionaries given by the BB-SPICE parser, via the the Python libSBML [34] module. Parameters with explicitly defined value are converted into SBML parameters whereas parameters which values depends on other parameters are converted into assignment. Translation of species definition with associated initial concentration is straightforward. Then, each reaction is converted into a SBML reaction. For each of them, reactant, products and modifiers are deduced from the netlist and the MathML formula of the equation rate is built from the reaction type and its associated parameters. Moreover, as degradation is not natively taken into account in SBML, an extra reactions have to be defined for species with non-zero degradation rate. Finally, SBML model does not involve generic fields for simulation directive. Thus, they are ignored

## Results

Performances of BB-SPICE are evaluated according to two criteria: the simulation accuracy and the computing time. For both criteria, the reference will be COPASI simulation of an equivalent SBML model.

### Simulation accuracy

**Fig 4:**
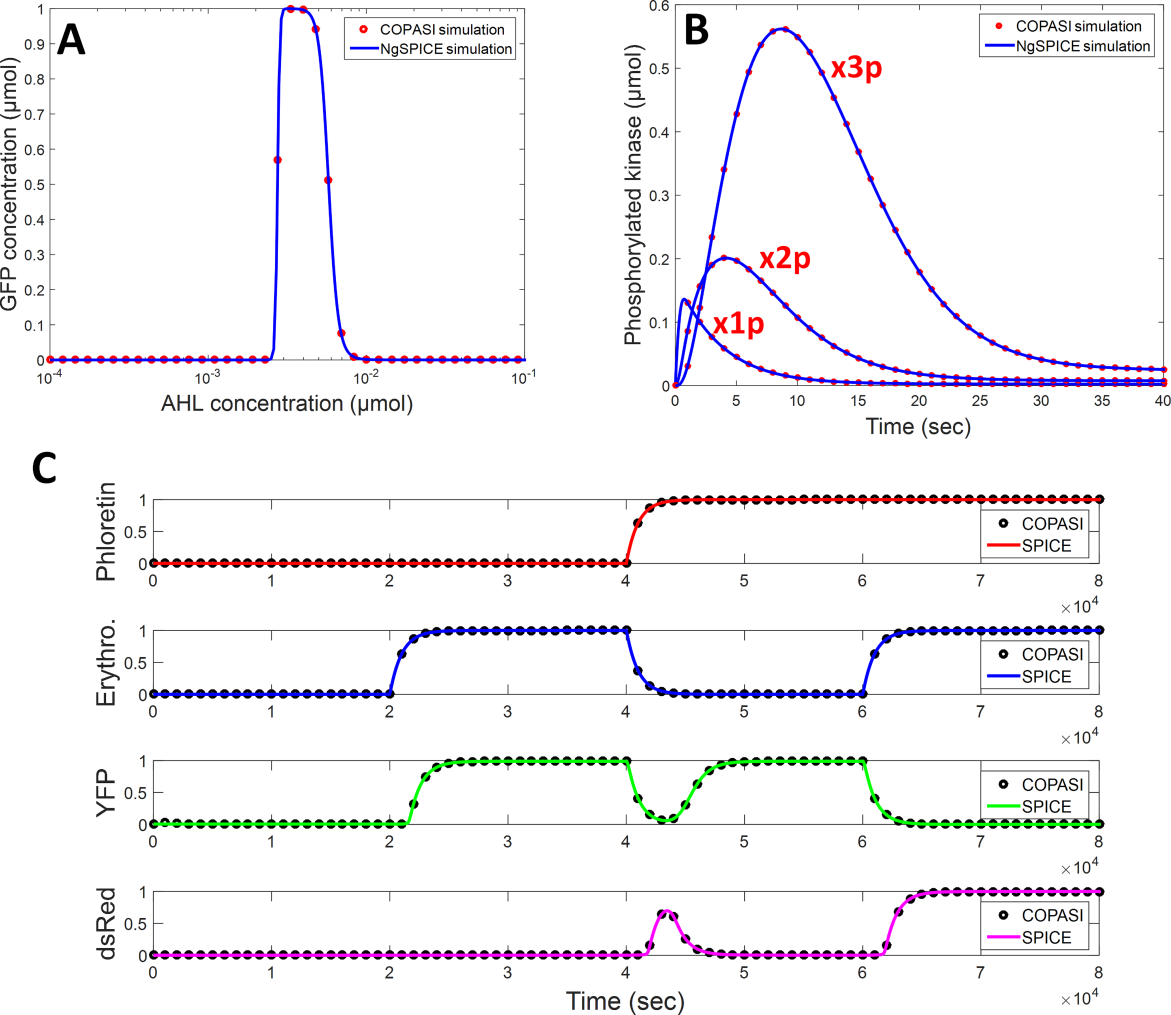
Comparison of simulation results obtained with a model written in BB-SPICE and, on the one hand, translated and simulated with NGSPICE and, on the other hand, translated in SBML and simulated with COPASI. The system modeled are a band detector (A), a kinase/phosphatase cascade (B) and a genetic half-adder (C).

First, we compare NGSPICE and COPASI simulations on a benchmark of 20 different biological systems, which covers all the mechanisms and the analysis capability of BB-SPICE. They are given in Table 3. Models have been written in BB-SPICE and converted both in NGSPICE and SMBL. Results are always in good accordance. To illustrate the purpose, simulation results obtained with three of them are given in Fig 4: the band-detector of Basu for which production of GFP occurs for intermediate concentration of AHL [35], a cascade of three kinase/phosphatase monocycle [36] and a mRNA-based half-adder with Erythromycin and Phloretin as input and YFP (sum) and dsRed (carry) as output [37]. The mean square deviation between results is very weak: 0.35% for the band detector, 30 ppm for the kinase/phosphatase cascade and 0.04% for the half-adder. The fact that COPASI and NGSPICE do not use the same integration method can explain this weak deviation.

**Table 3:** Benchmark of the biological systems used for the comparison between NGSPICE and COPASI.

Model name	Biological mechanisms										Analysis	Ref
	B	C	D	E	F	H	L	P	T	X		
Bio AND Gate	•						•		•		Pulse + Time Course	[38]
AND Feed-forward									•		Time Waveform	[39]
Band Detector (Basu)							•		•		DC sweep	[35]
Bistable reaction	•										Time Course + IC	[40]
Ca^2+^ oscillations	•					•				•	Time Course + IC	[41]
Circadian clock	•					•	•			•	Time Course + IC	[42]
Cdc2 / cyclin inter.	•	•	•							•	Time Course + IC	[43]
Diffusion					•						Time Course + IC	-
Ca^2+^ enzyme osc.		•		•		•	•				Time Course + IC	[44]
Birhythmic osc.		•	•							•	Time Course + IC	[45]
ERK Cascade				•		•					Time Course + IC	[36]
Feed Forward							•		•		Pulse + Time Course	-
Goldbeter Ca^2+^ model	•	•						•		•	Fix + Time Course	[33]
Half-adder	•						•		•		Truth Table + Time Course	[37]
Red. Prey-Predator			•							•	Fix + Time Course	[46]
Repressilator							•		•		Time Course + IC	[47]
Toggle Switch							•		•		Pulse + Time Course	[32]
uRNA amplifier	•						•		•		Transfer Function	[48]
uRNA logic func.	•						•		•		Pulse + Time Course	[48]
Enzymatic XOR				•			•		•		Truth Table + Time Course	[38]

List of the 20 biological systems that form the benchmark used to compare SPICE with COPASI on files generated from a common BB-SPICE netlist. For each, the type of the biological reaction involved in the model, the type of analysis and the reference in which the model has been published are given.

### Model conversion

The same comparison has been made with SBML models picked up in the BioModels Database [19] in order to validate the SBML to NGSPICE conversion. A benchmark of 25 models has been considered. It is described in Table 4 All the features described in the Methods section are covered by this benchmark. For all of them, simulation results obtained with SBML model on COPASI and with translated model on NGSPICE are in good accordance.

**Table 4:** Benchmark of the SBML models picked up from the Biomodels database, used for the validation of the SBML to NGSPICE translator.

BM#		Comp	Func	Para	Spec	Assi	Rules	Reac	Event	Ref
1	Nicotinic acetylcholine receptors	1	0	27	12	0	0	17	1	[49]
3	Mitotic oscillator	1	0	3	3	2	0	7	0	[50]
5	Cell division cycle	1	0	0	7	2	0	9	0	[43]
6	Cell division cycle (*reduced model*)	1	0	4	4	2	2	3	0	[43]
8	Cell cycle	1	0	3	5	2	0	13	0	[51]
9	MAPK Cascade	1	0	1	22	4	0	20	0	[52]
12	Repressilator	1	0	7	6	9	0	12	0	[47]
13	Calvin Cycle in plants	2	0	1	27	0	0	21	0	[53]
14	MAPK Cascade / Scaffold proteins	1	0	0	86	0	0	300	0	[54]
16	Circadian Clock	3	0	0	7	1	0	10	0	[42]
20	Squid axon	1	0	9	0	14	4	0	0	[55]
21	Circadian Clock	2	0	2	10	2	0	24	0	[56]
35	Genetic Oscillators	1	0	0	10	0	0	16	0	[57]
51	Carbon metabolism	2	0	0	18	7	0	48	0	[58]
55	Circadian Clock	1	1	65	13	0	0	32	0	[59]
60	Ryanodine receptors	1	0	0	4	1	0	3	0	[60]
65	Lac operon	1	0	23	9	0	0	16	0	[61]
73	Circadian clock	1	4	0	16	0	0	48	0	[62]
77	GnRH-induced LH secretion	1	0	2	8	1	0	5	1	[63]
88	Myosine phosphorylation	3	0	0	105	0	0	110	1	[64]
89	Circadian clock	1	1	78	16	0	0	36	0	[65]
120	TCell repectors	1	0	10	5	1	0	10	2	[66]
182	Intercellular signalling control	3	0	9	37	19	0	32	0	[67]
250	Controlled gene expressions	3	56	141	49	0	0	78	0	[68]
318	Rb-E2F switch	1	0	0	7	0	0	17	2	[69]

List of the 25 biological systems that form the benchmark used to compare SPICE with COPASI on files generated from a SBML file from the Biomodels database. For each of them, the identifier in the Biomodels database, the name of the reaction, the number of SBML structures (compartment, used-defined function, global parameters, species, assignment, rules, reactions and events) implemented in the model as well as the reference of the paper in which the model is published are given.

### Computation time

**Fig 5:**
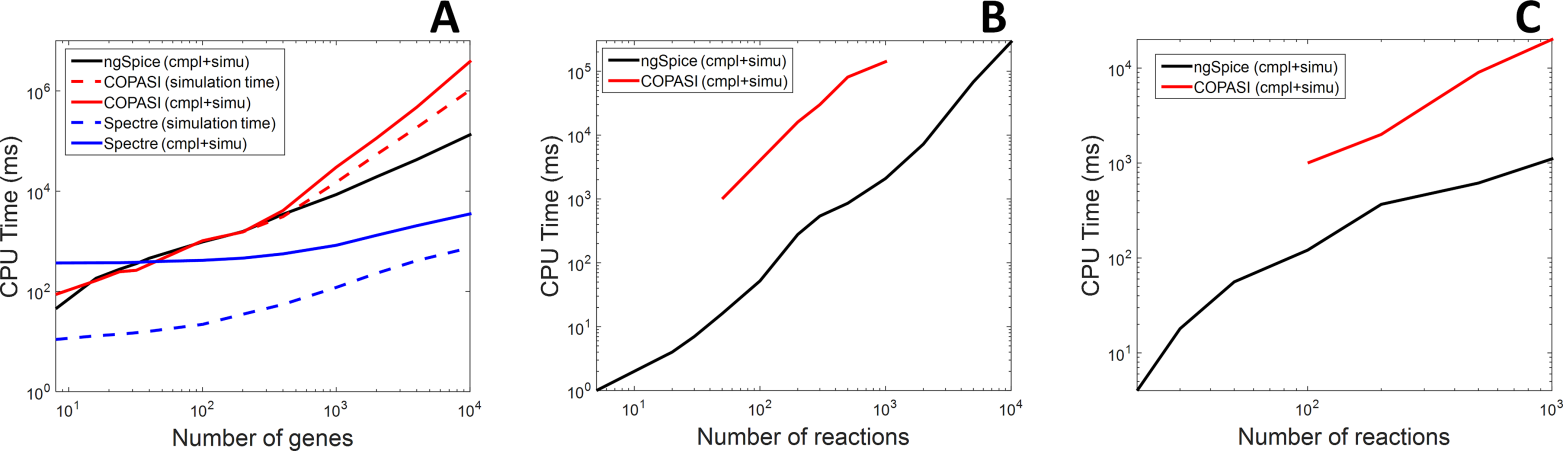
Comparison of computation time obtained with NGSPICE, COPASI and SPECTRE® for two different benchmarks of biological models. (A) is the transient simulation of gene regulator networks composed of variable number of genes. For SPECTRE® and COPASI, compilation time and simulation time are distinguished. (B) is the steady state simulation of linear metabolic pathways with a variable number of involved species. For COPASI, the range of tested value is reduced due to two limitations. First, the resolution of the CPU time measurement is the second. Thus, at least 50 reactions are required to obtain a significant value. Second, above 1000 reactions, the model is too huge to compilation failed due to memory errors. Finally, (C) is the steady state of metabolic pathways with 20 species and a variable number of reactions. Again, computation time measurement is not significant with COPASI under 50 reactions.

The second criterion used to compare NGSPICE and COPASI is the computation time. Two artificial benchmarks of large scale models have been generated. The first one, is composed of large scale oscillating gene regulatory networks (GRN) based on the principle of the repressilator [47]. The networks contains an odd number of genes going from 3 to 4999 genes repressing each. Taking mRNA into account, such models involves from 8 to 10000 reactions and species; a DNA transcription that synthesize an mRNA and the translation of this mRNA into the transcription factor for each gene + the mRNA and the reporter. Long-time transient simulations are carried out on these models. The simulation result, the compilation time of the model and the simulation time are monitored for three different tools: COPASI, NGSPICE and a commercial SPICE simulator optimized for multi-core processing, SPECTRE®. Results are given in the Fig 5A. CPU time for NGSPICE and COPASI are comparable for GRN with up to 500 genes. However, over this value, compilation and simulation time take off with COPASI while it remains linear with the number of genes with NGSPICE. With 10,000 reactions, the speed up offered by NGSPICE is about 30. Moreover, a speed-up of 40 can be observed between SPECTRE® and NGSPICE simulation time for large systems. Computation time for SPECTRE® is always dominated by the compilation step, which can hardly benefit from parallelization, unlike simulation. It should be mentioned that the compilation is only required once when the model is modified.

The second benchmark is made of models of random metabolic pathways. The pathways are characterized by two values: the number of involved *N* and the number of reactions *M*. Each pathway is composed of a linear path (*i*.*e*. the substrate of the *N*^th^ reaction is the *N*^th^ species and its product is the *N* + 1^th^ species) as well as several randomly drawn feedback and feedforward (reaction *N* + 1 to *M*). In addition, each reaction is catalyzed by one of the involved species in the pathway (randomly drawn) according to a Michaelis-Menten model. On each model, the steady state is computed. A comparison of computation time between NGSPICE and COPASI for linear pathways (*N* = *M*) is given in Fig 5B. The nodal analysis performed by NGSPICE is much more efficient that COPASI algorithm. A speed-up of 50 is observed for pathways with 50 to1000 species (pathways with more than 1000 species have not been tested with COPASI because models failed to compile) without any lost in term of accuracy: the steady state concentration obtained by both tools is comparable with a mean square error of less than 10 ppm. Second, pathways with 20 species and a variable number of feedback and feedforward reactions are simulated. Results are given in Fig 5C. The same tendency is observed, confirming the interest of using SPICE, especially for large systems. This result was predictable insofar SPICE has emphasis on integrated circuits, which are, by nature, very large scale systems.

### Models integration:A penicillin biosensor

**Fig 6:**
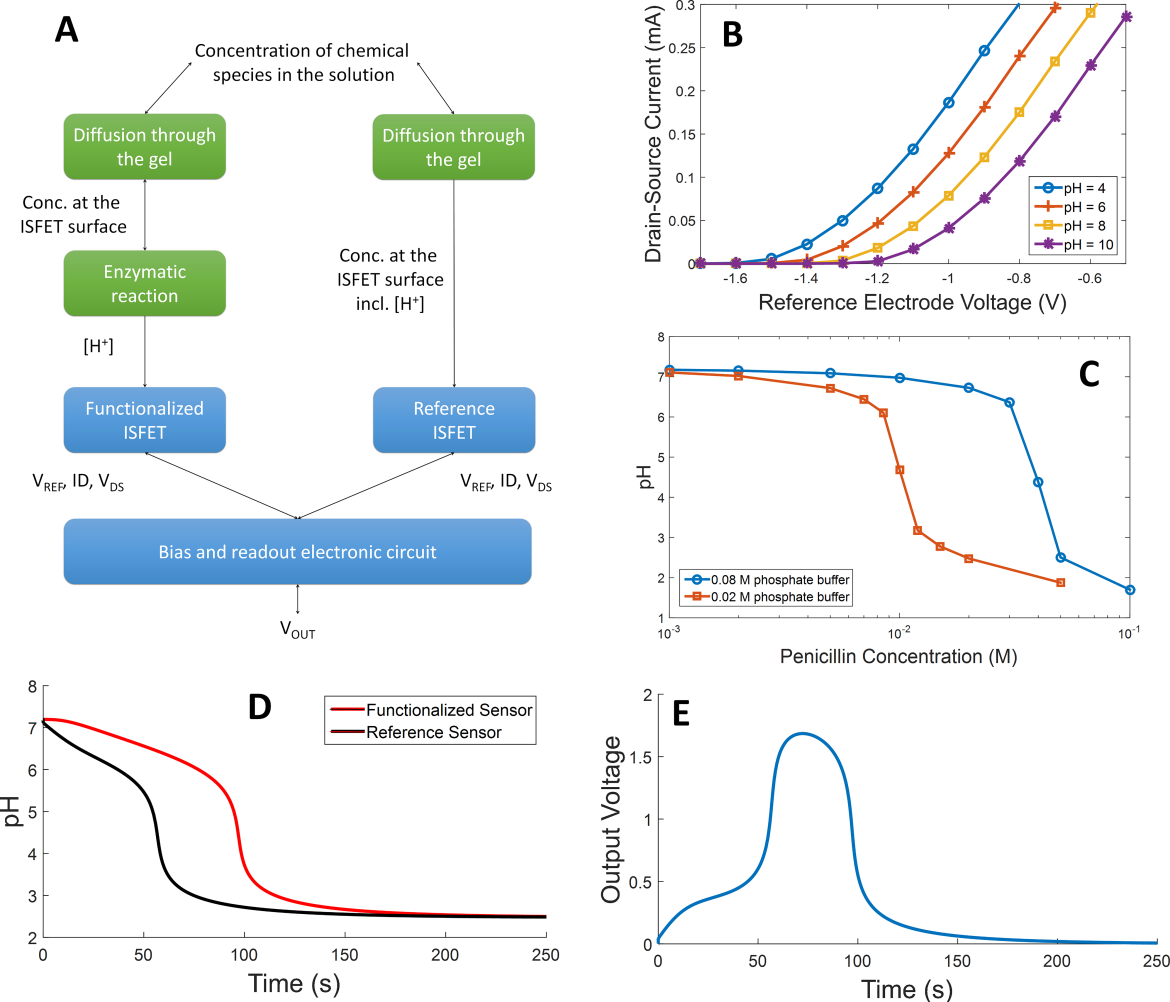
Results on the penicillin sensor. (A) is the overview of the building blocks of the model. Green blocks have been described in BB-SPICE whereas blue ones have been described directly in NGSPICE and integrated in the BB-SPICE netlist preceded by the ∃ character. (B) is a drain-source current vs reference electrode voltage characteristics of the ISFET alone. (C) and (D) are the simulation results for the biological part of the model alone. (C) is the variation of pH in the solution as a function of concentration of penicillin for two different phosphate buffer concentrations. (D) is the transient evolution of the pH on the gate of both ISFETs for a penicillin concentration of 0.05 mol/l. Finally, (E) is a transient simulation for the whole model (biological part, ISFETs, biasing and processing electronics). The evolution of the output voltage is given for different initial penicillin concentrations.

BB-SPICE paves the way for the modeling and the simulation of biosensors in a single language and environment. This purpose is illustrated on a penicillin sensor described by Caras et al. [70]. It is composed of two ion-sensitive field effect transistor (ISFET) [71]. The gate of one of them is coated with penicillinase and both are in contact with a reaction chamber which contains penicillin and a phosphate buffer via a permeable gel. At the surface of the functionalized ISFET, penicillinase catalyzes the hydrolysis of penicillin, releasing H^+^ ions that are sensed by the ISFET. A readout circuit converts the modification of the characteristics of the ISFET into an exploitable voltage. The building blocks of the model are described in Fig 6A. The biological part is composed of 16 chemical species (only 6 chemical species are involved in the system but 5 of them are instantiated three time to distinguish the concentration in the chamber and at the surface of each ISFET), one enzymatic reaction, 10 diffusion mechanisms through the gel membrane and 3 binding reactions for the phosphate buffer in the reaction chamber and at the surface of each ISFET. Martinoina’s model is used for the ISFET [72] and the readout circuit composed of standard electronic device only is the one described in [73]. Simulation results are given in Fig 6B to Fig 6E. Those results are in accordance with experimental data that can be found in the literature [73,74]. On the output response with an intermediate concentration of penicillin (Fig 6E) three phases can be identified. First, several penicillin molecules reach the penicillinase layer due to diffusion. This leads to the generation of H^+^ but the phosphate buffer plays its role and the pH variation is weak. Second, much more H^+^ ions are accumulated. They saturate the buffer and the pH decreases quickly. Finally, generated H^+^ ions diffuse back to the solution and to the reference ISFET. In the meantime, all the penicillin is consumed. At the equilibrium, pH in the chamber and on both ISFET is homogenous and the output voltage decrease to 0. If the initial concentration of penicillin is high, the first stage is too short to be observed. Conversely, if the initial concentration of penicillin is low, the buffer is never saturated and the pH does not change.

## Discussion

BB-SPICE is a powerful alternative to traditional modeling and simulation tools used in systems biology and synthetic biology (mostly SBML and COPASI). The writing of the model is less explicit than in SBML but much more faster and efficient, with condensed information recorded directly in a text file. The results described hereabove show that, in terms of accuracy, both approaches are equivalent and that the back-and-forth conversion between BB-SPICE and SBML is efficient. In terms of computation time, there is a significant gap in favor of NGSPICE in comparison with COPASI for the steady state calculation and for transient simulation of large systems. The speed up is significant enough to allow modeling and simulating much more complex systems than the one that could be handled by COPASI. Moreover, it can be pointed out that significant speed up (more than three orders of magnitudes) can be obtained with a commercial simulator (namely SPECTRE® from CADENCE®) optimized for multicore computation of large scale electronic circuits. Again, this speed up makes possible the study of more complex biological systems.

BB-SPICE and NGSPICE does not cover all COPASI capabilities. For example, it does not support flux balance analysis [75], elementary modes analysis [76] or stochastic simulations [77], which are useful tools for the theoretical study of metabolic pathways. Nevertheless, such analysis remain feasible by using the SBML model generated from the BB-SPICE netlist and COPASI or by post-processing on NGSPICE results. Conversely, it makes possible alternative analyzes that are widely used in electronics and may have potential to exploit in biology, *e*.*g*. small-signal (AC) frequency analysis around a steady state, noise sensitivity analysis or circuit optimization algorithms [7].

The performances of open-source simulation tools for electronic circuits are likely to increase over the next few years to adapt to the increasing complexity of the electronic circuits and devices. One of the most considered improvements is the deployment of simulation algorithms on GPU. CUSPICE already offers a GPU-compatible version of NGSPICE [78]. Unfortunately, this version does not support VCCS, which is widely used in our models. Nevertheless, there is no mathematical or technological lock against the integration of VCCS in CUSPICE. Alternatives to CUSPICE, such as TinySPICE [4], also exists and deserve to be investigated. As BB-SPICE generates SPICE-compatible netlists, changing SPICE simulator is straightforward. To optimize further, another outlook is to deploy directly the models of biological mechanisms as new devices hardcoded in NGSPICE or CUSPICE instead of using VCCS, as it is the case for electronic devices (diode, BJT transistors, MOSFETs).

Another interest of BB-SPICE standard is to make biological models directly compatible and connectable with electrical models. This point has been illustrated on the penicillin sensor. The model is described in a unique text file which contains the BB-SPICE model of biochemical reaction and a NGSPICE models for the ISFET and the biasing and readout circuit. Moreover, the compatibility of biological and electronic models is of great interest for the reuse of EDA tools in the field of synthetic biology, as it has been discussed in the introduction of this article.

As a conclusion, BB-SPICE is open-source and available for the biology community as well as for emerging engineering discipline at the interface with biology (synthetic biology, metabolic engineering design of biosensors, lab-on-chips etc). In its present version, it supports the most common biological mechanisms involved in a biological system. We wish to evolve it to fit as much as possible the needs of the community. In parallel, we hope that BB-SPICE inherit from future improvements of the open-source NGSPICE simulators both one the side of computational algorithms and on the side of optimization and parallelization on multi-core computers or on GPUs.
